# Observation of the Kibble–Zurek Mechanism in Microscopic Acoustic Crackling Noises

**DOI:** 10.1038/srep21210

**Published:** 2016-02-15

**Authors:** H. O. Ghaffari, W. A. Griffth, P.M. Benson, K. Xia, R. P. Young

**Affiliations:** 1 University of Texas at Arlington, 500 Yates St. Arlington, TX 76019; 2 Rock Mechanics Laboratory, School of Earth and Environmental Sciences, University of Portsmouth, Burnaby building, Portsmouth, PO1 3QL, UK; 3 Department of Civil Engineering and Lassonde Institute, University of Toronto, Toronto, 170 College Street, M5s3e3, On, Canada

## Abstract

Characterizing the fast evolution of microstructural defects is key to understanding “crackling” phenomena during the deformation of solid materials. For example, it has been proposed using atomistic simulations of crack propagation in elastic materials that the formation of a nonlinear hyperelastic or plastic zone around moving crack tips controls crack velocity. To date, progress in understanding the physics of this critical zone has been limited due to the lack of data describing the complex physical processes that operate near microscopic crack tips. We show, by analyzing many acoustic emission events during rock deformation experiments, that the signature of this nonlinear zone maps directly to crackling noises. In particular, we characterize a weakening zone that forms near the moving crack tips using functional networks, and we determine the scaling law between the formation of damages (defects) and the traversal rate across the critical point of transition. Moreover, we show that the correlation length near the transition remains effectively frozen. This is the main underlying hypothesis behind the Kibble-Zurek mechanism (KZM) and the obtained power-law scaling verifies the main prediction of KZM.

The spatio-temporal evolution of acoustic signals, a class of crackling noise^1^, is a direct result of failing atomic bonds during material fracture. Such signals, if properly interpreted, may be used to better understand the dynamics of rupture progress in the vicinity of crack tips over a broad range of scales and conditions^2^,^3^,^4^. During microcrack propagation, part of the stored energy near the crack tip is consumed in the breaking of molecular and atomic bonds, resulting in new crack surface area. The key to understanding the crackling process lies in the characterizing structure of the near-tip region of such microcracks, where stress amplitudes are large. Due to the microscopic size and high speeds encountered in the vicinity of the crack tip, direct measurements are difficult, and analysis typically relies on computational techniques^5^. Because of the large strains present near crack tips, nonlinear elastic and/or inelastic contributions must occur, and recent work^2^,^5^ suggests that non-linearity around the moving crack tip governs the rupture velocity. Specifically, the local hyperelastic or plastic zone around the moving crack tip enhances energy flow for stiffening systems, and reduces energy flow for softening systems, resulting in increases and decreases in the fracture velocity respectively^2^. Furthermore, investigations of “*slow*” cracks in gels demonstrate a link between the spatial energy flow around the rupture tip and the curvature of the tip^5^. This link is thought to be responsible for inaccuracies in linear elastic analyses that are commonly used in material science for simulating crack tip processes, with applications ranging from metal fatigue to earthquake nucleation. In all of the mentioned studies (but see^6^), the assumption is that cracks evolve under equilibrium conditions, and all crack tip processes satisfy an (quasi)adiabatic-equilibrium assumption. Under this assumption, the ramp time (the duration that the system approaches the critical point at which unstable crack growth proceeds) is at least of the order of the relaxation time of the system^7^,^8^,^9^,^10^. In this study we question this assumption by showing that fast processes (including many sub-excitations possibly due to lattice distortions or dislocations) involved in the structure of fast-moving crack tips induce a “frozen” regime where – for a very short time - the crack tip is out of equilibrium and the dynamics are very slow.

In this study we use a functional network method^11^,^12^ applied to acoustic emission (AE) data recorded during rock deformation and rock friction experiments (see methods section) to show that moving microcracks contain signatures of non-linearity. We discover that the onset of the non-linear stage prior to unstable failure coincides with the nucleation of kink instabilities, and we use “*network strings”* to visualize spatio-temporal evolution of these topological defects. For the first time, we show that emitted crackling noises hold the signature of the Kibble-Zurek mechanism (KZM)^7^,^8^,^9^,^10^ that provides an estimation of the defect density as a function of the traversal rate across a phase transition. In the current case, the “phase transition” is a transition between a strengthening and unstable weakening state during microcrack propagation. A key output of KZM relates faster ramp rates to higher defect density, as the result of a spontaneous symmetry breaking process. We measure real-time evolution of a first-order correlation function of the system (in network space) and verify the main prediction of KZM: namely the power-law scaling of the (frozen) correlation length with the ramp rate. Moreover, we show that the correlation length near the transition remains effectively frozen. This is the main underlying hypothesis behind the Kibble-Zurek mechanism^7^,^8^. In addition, using our laboratory datasets we illustrate that the adiabatic-impulse transition, as the core of the KZM hypothesis, can be used to infer an approximate weakening rate.

We analyse AE waveforms (the laboratory analogue of seismograms due to rock fracture or earthquake rupture) under different simulated depth (pressure) conditions and loading paths, and on two rock types: Westerly granite and Basalt from Mount Etna, Italy. (datasets Lab.EQ1, Lab.EQ2, Lab.EQ3 and Lab.EQ4 - see methods section and supporting information). We apply tools from the theory of complex networks to analyze emitted noises from microscopic cracks, where the acoustic time series recorded at each sensor is represented as a node [SI-section 1; refs 11,12]. These results allow us to develop an interpretation of recorded multiple acoustic-crackling signals involving a microsecond evolution of different dynamic crack tip phases as encoded in the network modularity (*Q*) which we refer to as “*Q-profiles*” (see methods section). This evolution can be broken down into three distinct phases (Fig. 1a,d,e): (1). The S-phase: an initial strengthening phase preceding the critical point at which point weakening and catastrophic failure begins; (2) the W-phase: a fast-slip or weakening phase; and (3) the D-phase: a slow slip or decelerating phase^11^,^12^,^13^. To better understand the S-phase and how the transition occurs across the critical point between S to W, we use the reciprocal of modularity (R = *Q*^−1^) profiles (i.e., “*R-profiles*”) which closely resemble dynamic stress profiles commonly used to characterize rock failure (Fig. 1). R-profiles are indicative of the dynamic stress changes due to a given cracking event. R_max_ corresponds to the critical point, where the failure occurs and fast-weakening begins.

In the following discussion, we describe the observation of “*defect*” formation prior to onset of the W phase. To proceed, we define the critical zone onset, R_c_, as the value of R at the time of the first impulse in the inverse of mean betweenness centrality 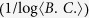 profile, where 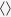 indicates the mean value of all nodes (Figs 2 and 3). In Fig. 2, we show 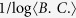 profiles for 6 acoustic events from our laboratory tests. For all events this profile is characterized by a narrow interval at the transition from the S to W phase. Later, we will show that the first impulse corresponds to the first nucleated defect in transition from S to W while the second spike indicates defect formation in an inverse transition (W to S).

In order to study the spatial variability of this impulse regime, we visualize the spatial evolution of the degree *k*_i_ of the *i*^th^ node, using polar coordinates 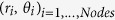 where *r*_*i*_ = *k*_*i*_ and *θ*_*i*_ indicate the position of the node which is fixed on the outer circumference of the cylindrical sample; Fig. 3c. We refer to these configurations as “*K-strings*”, and the normal vector of the *K*-strings at each node indicates the local direction of increasing or decreasing *k*_i_ with time. We evaluate the variation of *r*_*i*_ = *k*_*i*_ at each position (node) while we consider the temporal evolution of each single event (Fig. 3; Figs S5 and S6). In Fig. 3, we show that the onset of the impulse zone coincides with the folding of *K*-strings where the normal vectors are flipped at the onset of the non-linear regime and form a local domain that we refer to as a “kink” (Fig. 3d). Formation of domains in the course of the S-W transition results in a non-linear behaviour in the *S* phase of the *R*-profile. Since the trend of *R*(*t*) mirrors the mean value of *k* for all nodes <*k*(*t*)> (Fig. S4), we might also infer 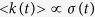. Given this observation, it is clear that the non-linearity of stress is well-connected to nucleation of these network kinks.

As emphasized by Polyakov^14^ in the context of string theory, crumpled stings are analogous with the Heisenberg paramagnet while undulations destroy long-range order in surface normals^14^,^15^. These topological defects are local defects in initially ordered structures and can be removed by global collapse of K-strings and local bending or twisting around the defects cannot remove them (i.e., they are topological defects)^16^,^17^. To analyze deformation of *K*-strings, we map them onto simplified spin-like chains where for each node we assign 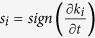 and then *s*_*i*_ = ±1. With this mapping, defects represented with negative *s*_*i*_ indicate flipped, inward-pointing normal vectors (spins). We have simplified a true 3D configuration of acoustic networks by assigning one component per each node (up or down) –see Fig. S7. A double kink separating zones with up and down spins functions as a locator for the change from one ground state (S-phase) to another degenerate ground state (W-phase)^17^,^18^,^19^.

The critical point <*k*>_max_ is defined when 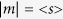 approaches its minimum value (Figs 4 and 5b). Here, *m* is the *order parameter* of the K-strings and the transition from S to W (and vice versa) occurs continuously. A stable disorder phase *m* ≈ 0 precedes the onset of the *S*-phase, and the system of nodes is forced from a disordered state (prior to *S-phase*) to an ordered state (*S*). This happens continuously and is a symmetry-breaking transition. The disordered state is a symmetric state and in the ordered phase the order parameter (*m*) chooses one direction; for the *S*-phase the mean direction is positive (↑ outward), whereas for the *W*-phase, the mean direction is negative (↓ inward). Approaching <*k*>_max_, the system rapidly transverses a temporary-unstable symmetric state. The reason lies in the fact that the symmetric state is not the state of minimum energy and that in the process of evolving toward the ground state, the symmetry of the system has been broken^18^.

To proceed, we calculate a correlation function for the K-strings as they approach <*k*>_max_ (Fig. 4a,b). We can fit a correlation function such as 
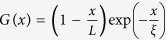
 where *L* is the total number of nodes, *x* is distance, and *ξ* is the correlation length^19^,^20^. Correlation length *ξ* is the cut-off length of where for distances shorter than *ξ* the correlation function G(x) can be fitted. For a fully ordered state a triangular function (where *ξ* → ∞) is given by the green line in Fig. 4c.

Interestingly the correlation length becomes essentially frozen as <*k*>_max_ is approached, as shown in Fig. 4d for crackling event #255. More examples shown in Fig. 5 indicate that the results are universal for the recorded acoustic events. This means as the system approaches the critical point at a finite rate, and after some point in time the correlation length cannot maintain its equilibrium value and a transition occurs with *ξ* much smaller than the system size. This correlation length sets the mean finite-size of the final domains. The formation of domains as well as the observation of frozen correlation length at the critical point of our acoustic networks indicate the existence of out-of-equilibrium mechanism that is well-described by the Kibble-Zurek mechanism (KZM, see Methods section)^7^,^8^,^9^,^10^.

The core idea behind the KZM is that near the phase transition, freezing of the correlation length is unavoidable. Based on this theory, the resulting density of defects left behind by continuous transitions is dependent on the rate at which the critical point is traversed, and the rate with which the system can adjust, defining the relaxation or healing time of the system. This mechanism is reflected in the density of defects and the “*freeze-out*” time which scales with ramp-rate. To test the KZM’s defect density prediction, we carefully measured the number of flipped nodes in the vicinity of the critical point where correlation length is frozen (Fig. 6).

A key output of our analysis is that the number of defects (i.e., flipped nodes at the final state) is larger when the local ramp rate 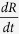 is faster (Fig. 6a). We can fit a power-law scaling as: 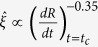 (Fig. 6a), where 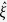 is the frozen correlation length and 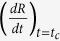 is the ramp rate (Figs S4–6 and S10). To determine the ramp rate, which is analogous to the local loading rate prior to the nucleation of kinks, we measure the slope of *R*(*t*) (Fig. 3b). The exponent of ∼*0.35* ± *0.06* obtained by fitting the laboratory data is in agreement (within experimental error) with the theoretical value *ν/(1* + *νz)*  ≅  0.34 where, for the 2d classical Ising system, *ν* ≅ 1 and *z* ≅ *2.1*^19^,^21^. Here, the parameters *ν* and *z* are spatial and dynamical critical exponents (see Methods section).

Furthermore, we can evaluate the time-reversal transition from the W to S phase while we approach to the critical point from right (in other words, if we heal or reverse the failure process). Approaching from the left (S-phase) or right (W-phase; time reversal or healing scenario) to the critical point *λ*_*c*_ results in slightly different characteristics of the defect density (Fig. 5b). The rate of the S-ramp (linear strengthening rate or ramp rate) for most of the recorded events is higher than the rate of W-ramp (i.e., linear weakening rate). Next we estimate the linear weakening rate. Using the KZM scaling law for defects, we obtain (Supplementary Information): 
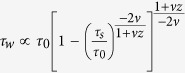
, in which *τ*_0_ is determined by the microscopic details of the system; *τ*_*s*_ and *τ*_*w*_ are the time characteristics of ramps in the S and W phases, respectively. Therefore, the linear weakening rate is given by: 
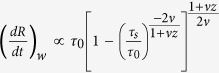
. A faster local S-ramp inversely scales with weakening rate. This is an important result since it has been shown that weakening rate is correlated with the global rupture velocity of cracks^3^,^12^,^22^. To verify this prediction, we measured the rate of weakening from R profiles 
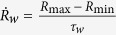
. As shown in Fig. 6b, our measurements confirm the aforementioned prediction.

## Discussion

We have shown, for the first time via laboratory data, the evolution of propagating microcracks over the duration of only a few microseconds derived from multiple AE event data. We mapped acoustic excitations from crackling events to complex networks and then further to spin-like chains. Applying these tools in a novel new way to AE data, we elucidated the transition to from a precursory strengthening phase to a phase of rapid weakening and unstable crack growth. We demonstrated a real-time probe of evolving ground state of the system via acoustic (phononic) excitations and then we accessed the strongly non-equilibrium dynamics directly, rather through its aftermath. We illustrated that as we force our system to cross the transition at a finite velocity, different regions of the system will choose different minima of the free energy. This leads to the appearance of topological defects. Using an analogy with the Ising ferrormagnet model, we discovered that topological defects correspond to the appearance of regions where the nodes form domains pointing either up or down.

As we described, flipped nodes induce a degree of non-linearity (inelastic behaviour) in dynamic stress profiles, and dislocations are good physical candidates for such defects in crystalline solids. Far from the critical point where the system is still coherent, thermal activation of dislocations occurs adiabatically; however rapid-traversing to the failure point violates this assumption. As another perspective to support nonlinearity of acoustic emission waves, we can assume that a *K*-string is a classical vibrating string (i.e., harmonic or anharmonic oscillators) where “folding” adds normal modes to the oscillator^23^,^24^. Assuming a string with the fixed boundaries at the end of elastic string has *n* normal modes, the boundary value problem of solving the wave equation results in: 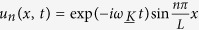, where 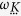 is the frequency of an excitation of wave-vector *K*, *L* is the fixed length of the string (number of nodes), and *u*_*n*_(*x*, *t*) represents the deflection of the string which satisfies the boundary condition. The fundamental mode *n* = *1* for our network-strings is a fully-ordered state. Each flipped node is an analogy with phononic (or generally bosonic) excitation. We might call them “*nodons*” to specify excitations on network-like structures. For a given wave-vector (*K*), the string could have *n*_*K*_ nodons (Fig. S12). With this description, we support the previous speculation on the non-linear nature of acoustic waves emitted from different sources^25^,^26^.

While our primary focus in this study was to characterize events with the definite continuous S-W transition, we have also recognized events with an abrupt change in the order parameter that is characteristic of a first order transition (Fig. S18). Further study is needed to explore more features of first-order “laboratory earthquakes”. In addition, analysis of 3D acoustic networks by mapping them to spin models will present a unique picture of the evolution of microcracks under true 3D stress-fields. It would be interesting to study the transition from fast-weakening phase to the next phase and monitor the evolution of nodes’ states under a true 3D acoustic networks (such as^12^) where this transition defines the crack-like or pulse-like nature of rupturing^27^. Another interesting study could focus on the dynamics of annihilation of flipped nodes and its effect on self-healing pulses. Finally, our results could be extended in the study of dynamics of frictional interfaces where dissipation of energy is coupled with the variation of contact areas^13^ as well as the study of stick–slip motion with the formation of kinks and antikinks^26^.

## Methods

### Laboratory Procedures

We use four sets of recorded acoustic emissions (labeled as Lab.EQ1, 2, 3 and 4) from Westerly granite and Basalt rock samples (most of the analyzed events are precursor rupture fronts). The Lab.EQ1 and 2 are the recorded multi-stationary acoustic waveforms from evolution of frictional rock-interfaces of Westerly Granite samples. The interfaces were in dry conditions with smooth (saw-cut) and naturally rough surfaces, respectively^28^. The evaluated events are from different stages and position and are not limited to particular stage of the tests. The Lab.EQ3 is the fast-loading experiment on a cylindrical sample of Westerly Granite (~10^−5^ s^−1^) at 50 MPa confining pressure (approximately 2 km), which is about an order faster than Lab.EQ1 and 2^29^ Lab.EQ4 are events from Basalt samples; described in^30^. The global loading rate was 10^−6^ s^−1^. In all of the above experiments, we reordered amplified events using 16 to 18 sensor networks in both short (discrete events) and long timescale recorders (AE records). The resolution of each recorded interval during the life-time of a waveform was ~20–100 ns.

### Networks of Acoustic Emission Waveforms

The concept behind studying each single acoustic excitation event – in this study – is to characterize sub-events involved in the course of just a single AE event. A big avalanche is composed of many smaller components, which trigger one another^1^. To study each acoustic event, we use functional network theory to analyze multiple recorded waveforms.

To evaluate reordered multiple acoustic emissions (multiple time series for a single event), we use a previous algorithm on waveforms from our reordered acoustic emissions^4^,^11^,^12^. The main steps of the algorithm are as follows^11^:

1. The waveforms recorded at each acoustic sensor are normalized by the maximum value of the amplitude in that station.
2. Each time series is divided according to maximum segmentation, in a way that each segment includes only one data point. The amplitude of the *j*th segment from the *i*th time series (1 ≤ *i* ≤ *N*) is denoted by *u*^*i,j*^(*t*) (in mV). *N* is the number of nodes or acoustic sensors.
3. *u*^*i,j*^(*t*) is compared with *u*^*k*,*j*^(*t*) to create links between the nodes using the following methodology: If 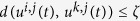 (where 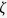 is the threshold level discussed in the following point) we set 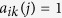, otherwise 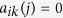 where *a*_*ik*_(*j*) is the component of the connectivity matrix and 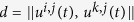 is the employed *similarity metric*. The employed norm in our algorithm is the *absolute-norm*. With this metric, we simply compare the amplitude of sensors at each time-step.
4. Threshold level (*ζ*): To select a threshold level, we use a method^4^,^11^ that uses an adaptive threshold criterion and is stable for a range of deviations from *ζ*. The result of this algorithm is an adjacency matrix with components given by 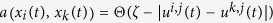 where Θ(...) is the Heaviside function.

In general, the modularity of a network measures the degree of division of that network into modules (clusters): if a network has high modularity, the strength of connections in individual modules is strong, whereas the strength of connections between modules is not. The network’s modularity characteristic is addressed as the quantity of densely connected nodes relative to a null (random) model^31^. The modularity is quantified using the *Q-profile*, and is the result of some optimization of the cluster structure of a given network. The modularity *Q* (i.e., the objective function) is defined as^23^:


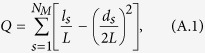


in which *N*_*M*_ is the number of modules, 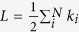, *l*_*s*_ is the number of links in module *s* and 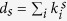 (the sum of node degrees in module *s*).

Then, in each time step during the evolution of the waveforms (here over observation windows of ~400 μs), we obtain a Q value. The temporal evolution of Q in the monitored time interval forms the Q-profile.

This network algorithm can be explained in the context of space-correlation methods. We can define a similar measure to a time-windowed correlation method^32^ where the inner product is replaced with a Heaviside function. Let us consider a sequence of nodes over a certain time step. The space-windowed correlation is given by^32^:


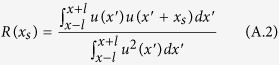


where the space-window of length L = 2*l* is centered at length *l*, *x*_*s*_ is the space shift used in the cross correlation, and the amplitude of each node is *u*(*x*). Here the employed norm is the inner-product and can be replaced with another norm:


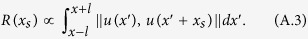


Summing over all space-shifts and replacing the norm with our similarity metric we get:


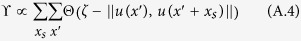


ϒ is proportional to density of links determined by the similarity metric used to construct links between nodes represented by pairs of amplitudes 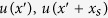.

### Kibble-Zurek Mechanism

The idea behind the KZM is to compare the relaxation time (or healing time of the system in equilibrium) with the timescale of change in the control parameter (ε). Assuming a linear change of control parameter in the vicinity of the critical point ε(*t*) = *t*/*τ*_s_, where *τ*_s_ is the ramp time in S-phase. The relaxation or healing time we consider is an equilibrium (quasi-static) condition: 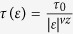 and *vz* = *μ*. This determines the reaction time of the order parameter. Here, *ν* and *z* are spatial and dynamical critical exponents, and *τ*_0_ is a characteristic timescale^8^,^9^. The system can adiabatically follow the change imposed by the local stress ramp if the relaxation time characterized by *τ*(ε) is outside the interval set by the “freeze-out” time 
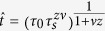
 centered around the transition point (see Fig. S3). The correlation length will effectively remain fixed (i.e., “freeze”) at time 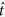 before reaching the critical point. The correlation length is given by^8^,^9^,^20^: 
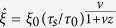
, where *ξ*_0_ is a characteristic correlation length.

Topological defects are formed with the density of one defect fragment per domain. An estimate for the resulting density of topological defects is given by^8^,^9^: 
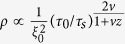
. In the frozen phase, one can define an effective control parameter 
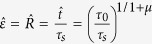
 21,33. By plotting events in 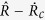 space in which 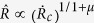, we obtain *vz* = *μ*. Then, we can estimate *ν* from 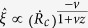 where we measure the frozen correlation length for the given event with the rate of transition 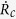. This procedure leads to estimates of *ν* ≅ 1 and *z* ≅ 2.1 which agrees with the mean-field approximation of the scaling coefficients of the 2D Ising-model (Z_2_ symmetry breaking^19^,^21^). To verify the KZM prediction for the scaling exponent, we analyzed many events for which the order parameter changes continuously (Fig. S15–17).

## Additional Information

**How to cite this article**: Ghaffari, H. O. *et al.* Observation of the Kibble–Zurek Mechanism in Microscopic Acoustic Crackling Noises. *Sci. Rep.* **6**, 21210; doi: 10.1038/srep21210 (2016).

## Supplementary Material

Supplementary Information

## Figures and Tables

**Figure 1:**
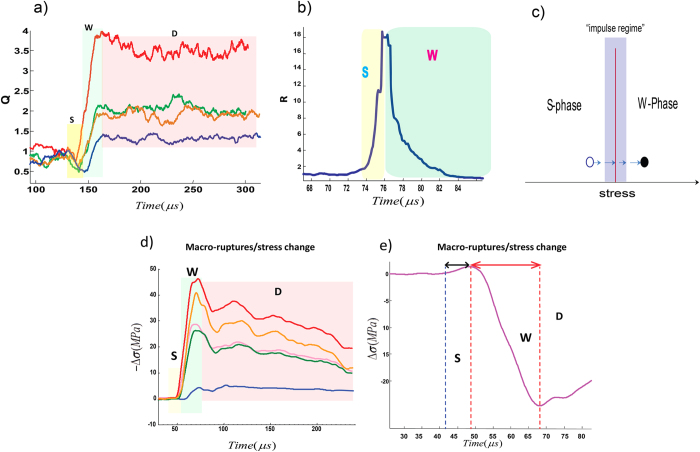
Q-profiles representing dynamic crackling noises. (**a**) Three main stages of typical acoustic crackling noises as shown in normalized Q-profiles: S-W-D phases correspond with strengthening, weakening and decelerating stages, respectively. (**b**) A typical -R profile as generated from dataset Lab.EQ1. **(c)** A schematic representation of impulse zone in transition from S to W phase. (**d**) Five recorded stick-slip events with (dynamic) strain gauges measurements in centimeter scales rock-interfaces^12^,^29^. (**e**) An event from (**d**) where stresses dynamically drop about 25 MPa during a stick-slip experiment.

**Figure 2:**
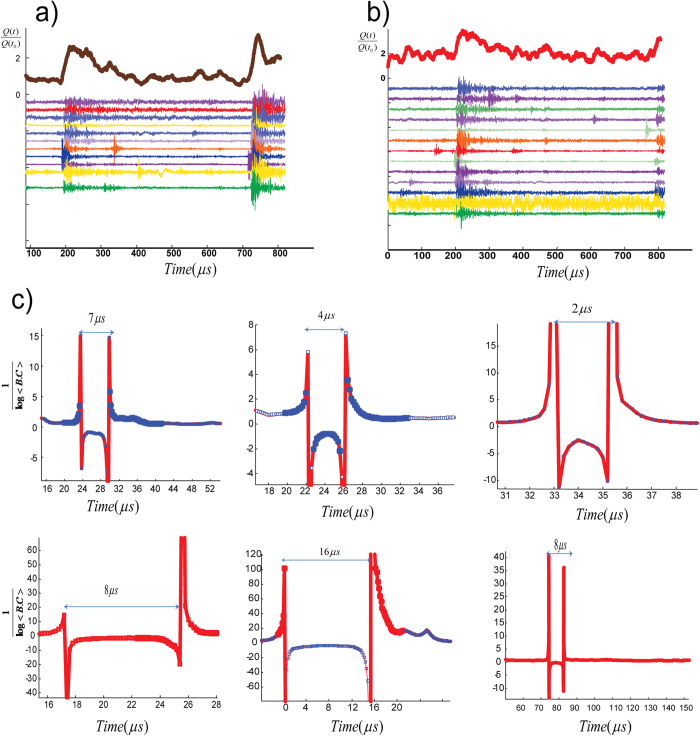
(**a**,**b**) Two typical acoustic emission events from cracking Granite samples (from Lab.EQ3). We have shown scaled recorded acoustic waveforms in ~800 μs and the corresponding normalized Q(t). (**c**) Different events from our tests with the signature of inverse of mean betweeness centrality which shows divergence of the parameter in vicinity of the nucleation zone. Based on the resolution of our measurements, the total time of order-disorder-order transition sequences stretches between 0.5–16 μs

**Figure 3:**
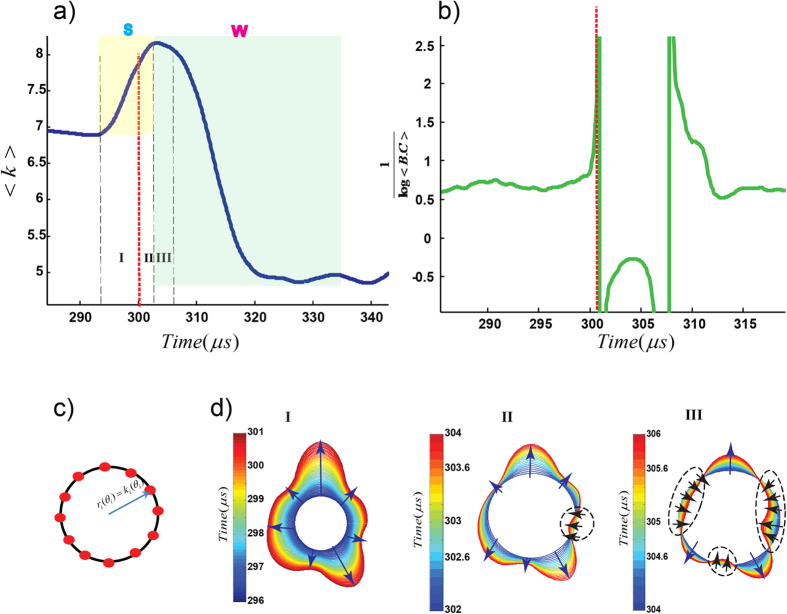
Nucleation of kinks and formation of domains. (**a**) The mean number of edges (<k>) versus time for event #24 from Lab.EQ4. (**b**) Transition to nucleation zone is imprinted in diverging the inverse of mean betweeness centrality (B.C). (**c**) Schematic representation of sensors location (red filled circles) where the radius of the ring is proportional with node’s degree (**d**) We have shown accumulated 2D spatio-temporal patterns of nodes’ degree in the polar system for each time interval as in panel (**a**). Transition from the linear stage (1) to the non-linear regime (2 + 3) is indicated by the onset of local defects (black arrows), inducing formation of pair-kinks. In the example reported, there are four major defect-zones. The arrows are normal to strings and crumpled strings destroy long-range order in string normal. We have shown more examples in Figs S5 and S6.

**Figure 4:**
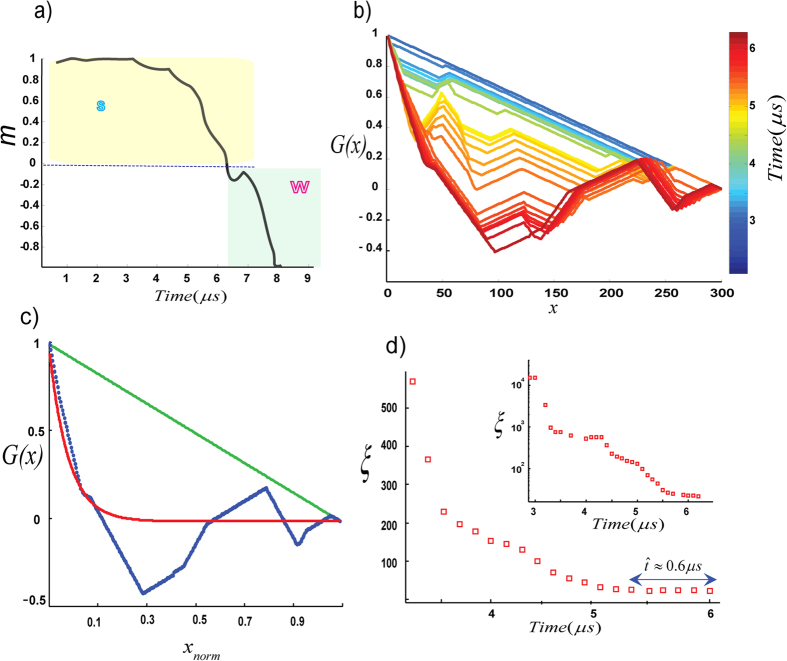
Continuous Phase Transition from S to W. Results from a cracking noise #255 from Lab.EQ4 with mapping on the Ising-chain (**a**) The real–time order parameter *m* versus time. (**b**) Real-time cross-correlation function. Very close to the transition point in red, we can see nearly overlapping patterns of G(x) indicating a frozen zone of correlation length (**c**) cross-correlation function versus the normalized distance. For fully ordered state a triangular function (fully coherent) is given by green line. Approaching transition point, we can fit a correlation function (red line), to determine correlation length. For this event, the frozen correlation length is 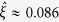. Blue points are the experimental measures of the correlation function. Before normalization, we had L = 300 nodes and 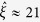 nodes. Approaching the critical point, the correlation length becomes frozen as shown in (**d**). For this event, the correlation length is roughly constant for ~0.6 μs. See supplementary Figures S13–S15 for more examples.

**Figure 5:**
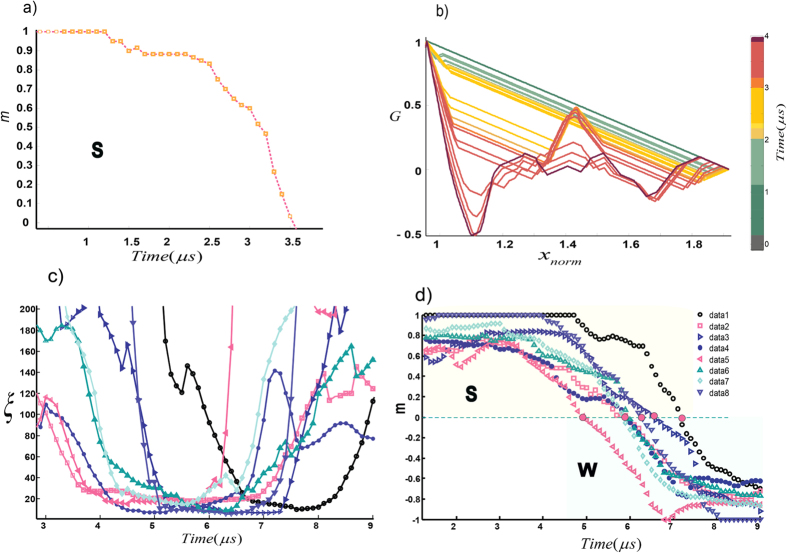
Real-time correlation length of Acoustic-Networks. (**a**,**b**) The real-time evolution of the order parameter in the S-phase and the corresponding pair-correlation for a dry-cracking event from Lab.Eq3. The fully coherent system has a triangular shape. 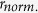 is the normalized distance between nodes. For early time steps (t < 3 μs), thermal activation of kinks does not destroy the long-range correlation which spans the whole system (**c**,**d**) When the system approaches the critical point, the correlation length ξ is effectively “frozen” as shown by the roughly horizontal portions of the curves in C. The size of the network system was 300 nodes and for the acoustic-networks far-beyond the critical point a long-range correlation length is assigned, i.e., the coherence spans the whole system.

**Figure 6:**
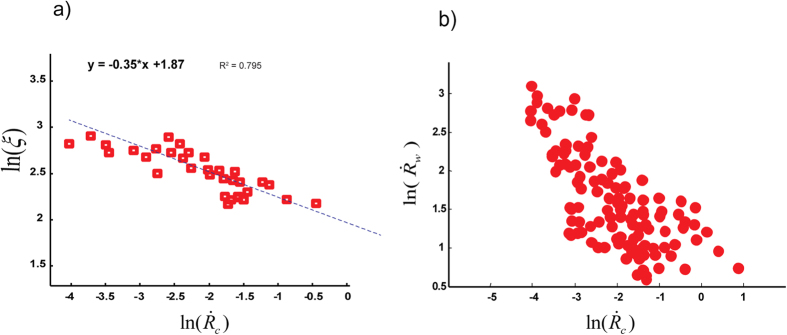
Dependency of frozen correlation length (kink density) on ramping rate. (**a**) Events with faster transition to their critical points induce higher defect density (i.e., shorter correlation length). Here we show typical rupture fronts from Lab.EQ3 and the size of the network is 300 nodes. We obtained *b* ≈ 0.35 in 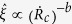 (dashed line), in agreement with the mean-field model prediction (also see Figs S14–S16) (**b**) effect of local stress-ramp rates on the fast-weakening regime: Events with faster weakening rate scale with slower local ramp rate. This is illustrated here via a *log-log* plot of the normalized rate of weakening 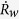 observed as a function of ramp-rate (events from Lab.EQ4).
