# Supplementary material for: Observation of the Kibble–Zurek Mechanism in Microscopic Acoustic Crackling Noises
Source: Sci Rep. 2016 Feb 15;6:21210. doi: 10.1038/srep21210 (PMC4753415; doi:10.1038/srep21210)
Supplement: Supplementary Information [file srep21210-s1.pdf]

Supplementary Information (SI)

Observation of the Kibble–Zurek mechanism in Microscopic Acoustic Crackling Noises

H.O. GHAFFARI [1 (A)] , W. A. GRIFFTH[1] ,P. BENSON[2],K.XIA[3] , R.P.YOUNG [3]

[1] *The University of Texas at Arlington, 500 Yates St. Arlington, TX 76019*

[2] *Rock Mechanics Laboratory, School of Earth and Environmental Sciences, University of Portsmouth, Burnaby building, Portsmouth, PO1 3QL, UK*

[3] *Department of Civil Engineering and Lassonde Institute, University of Toronto, Toronto, 170 College Street ,M5s3e3,On, Canada*

In this Supplementary Information, we provide further results and discussions on the proposed ideas in the main text.

1. Mapping Multiple-Acoustic Excitations to Network Space

To evaluate recorded multiple acoustic emissions (multiple time series for an occurred event), we use a previously published functional network algorithm on waveforms from our reordered acoustic emissions [1-3]. The main steps of the algorithm are as follows [1]:

(1) The waveforms recorded at each acoustic sensor are normalized to the maximum value of the amplitude in that station.
(2) Each time series is divided according to maximum segmentation (i.e., each segment includes only one data point). The amplitude of the jth segment from ith time series ($1 \le i \le N$) is denoted by $x^{i,j}(t)$ (with units of mV). N is the number of nodes or acoustic sensors. With considering the length of each segment as a unit, we considers the high temporal resolution of the system's evolution, smoothing the raw signals with 20-40 time windows (~400-800ns).
(3) $x^{i,j}(t)$ is compared with $x^{k,j}(t)$ to create an edge among the nodes. If $d(x^{i,j}(t), x^{k,j}(t)) \le \zeta$ (where ζ is the threshold level discussed in the following point) we set $a_{ik}(j) = 1$ otherwise $a_{ik}(j) = 0$ where $a_{ik}(j)$ is the component of the connectivity matrix and $d = \left\| x^{i,j}(t) - x^{k,j}(t) \right\|$ is the employed "similarity metric". With this metric, we simply compare the amplitude of sensors in the given time-step.
(4) Threshold level (ζ): To select a threshold level, we use betweenness centrality (B.C.)-see Eq.1 below for the definition of this property. In [4], we showed that selecting the threshold level by using the minimum variation of B.C versus ζ is equivalent to finding the most stable structures in the networks.

Each node is characterized by its degree k_i representing the number of links connected to that node, and its betweenness centrality (B.C) [5]:

$$B.C_i = \frac{1}{(N-1)(N-2)} \sum_{\substack{h,j \\ h \ne j, h \ne i, j \ne i}}^{N} \frac{\rho_{hj}^{(i)}}{\rho_{hj}}, \qquad (1)$$

in which ρ_{hj} is the number of shortest paths between h and j, and $\rho_{hj}^{(i)}$ is the number of shortest paths between h and j that pass through i.

In general, the modularity of a network measures the degree of division of that network into modules: if a network has high modularity, the connectivity in individual modules is strong, whereas the connectivity between modules is not. The network's modularity characteristic is addressed as the quantity of densely-connected nodes relative to a null (random) model. The

main diagnostic in this work is the *Q-profile*. The modularity is the result of some optimization of the cluster structure of a given network. The modularity Q is defined as [6]:

$$Q = \sum_{s=1}^{N_M} [\frac{l_s}{L} - \left(\frac{d_s}{2L}\right)^2], \qquad (2)$$

in which N_M is the number of modules (clusters), $L = \frac{1}{2}\sum_{i}^{N} k_i$, l_s is the number of links in module s and $d_s = \sum_i k_i^s$ (the sum of node degrees in module s).

We use the Louvian algorithm [7] to optimize Eq.2, which has been used widely to detect communities in different complex networks. Then, in each time step during the evolution of waveforms (here over observation windows of ~200 µs), we obtain a Q value. The temporal evolution of Q values in the monitored time interval forms the Q-profile. Three main stages for a single recorded acoustic excitation event are (Fig.S1)[1-2,8]: (1) S-phase: main deformation phase in the form of initial strengthening (2) W-Phase: a fast-slip or weakening phase , and (3) D-phase: a slow slip or decelerating stage (Fig. 2a). Comparing the evolutionary phases of micro-cracks to dynamic stress change from macro-sliding events (recorded by strain gauges) reveals similar time characteristics and trends (Fig.S1b-[1,9-10]). In using the reciprocal of Q-profiles (which we refer to as R-profiles), we magnify the initial strengthening phase.

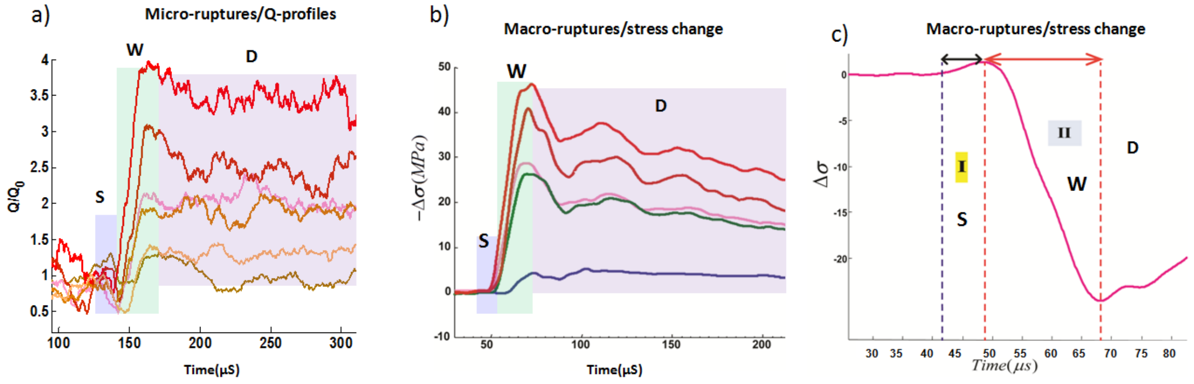

Figure S.1. (a) Main evolutionary stages (i.e.,S-W-D) of cracking acoustic excitations from **microscopic cracks** coincides (b,c) with (dynamic) strain gauges measurements from **macro stick-slip** of centimeter scales rock-interfaces [3].

2. Brief review of Experiments

Lab.EQ1: a saw-cut fault, which is embedded in a cylindrical rock sample, is perturbed slowly with a strain rate control feed-back. The detailed experimental results have been reported in [9]. Our main data set includes the recorded discrete and continuous waveforms (i.e., acoustic emissions-AEs) using 16 piezoelectric transducers from a saw-cut sample of Westerly granite under triaxial loading [9]. The saw cut was at a 60 degree angle and polished with silicon carbide 220 grit (Fig.S.2). Each triggered event had a duration of 204.8 μs (recorded at 10 MHz), while the three main stick-slip events occurred. The experiment was servo-controlled using an axial strain rate of $5\times10^{-6}s^{-1}$ ($\sim 10\mu m/s$ as the loading rate). The confining stress was maintained at 150 MPa for three reported main stick-slip events, producing 109 located- rupture fronts events.

Lab.EQ2: The second data set (LabEQ2) are the results of the two main cycles of loading – unloading (stick-slip) of Westerly granite on a preexisting natural fault by loading at constant confining pressure. A natural rough fault was created using a triaxial loading system at the constant confining pressure of 50 MPa and with acoustic emission feedback control [10].

Lab.EQ3: The third data set (LabEQ2) slightly faster loading of intact Westerly granite rock samples with similar loading condition (confinement loading) of Lab.EQ1 and 2. The details of the experiment have been reported in details in [11].

Lab.EQ4: Samples of basalt from Mt. Etna volcano (a basalt rock of approximately 3.8% porosity) were deformed using a standard triaxial deformation apparatus installed at University College London (UK). Cylindrical samples 40 mm in diameter and 100 mm in length were isolated from a confining medium (silicone oil) via an engineered rubber jacket containing inserts for mounting piezoelectric sensors in order to detect AE events. AE event signals (voltages) are first pre-amplified 40 dB, before being received and digitized at 10 MHz sampling rate (as well as Lab.EQ1,2,3). The sample was dry. The details of the test can be found in [12-13].

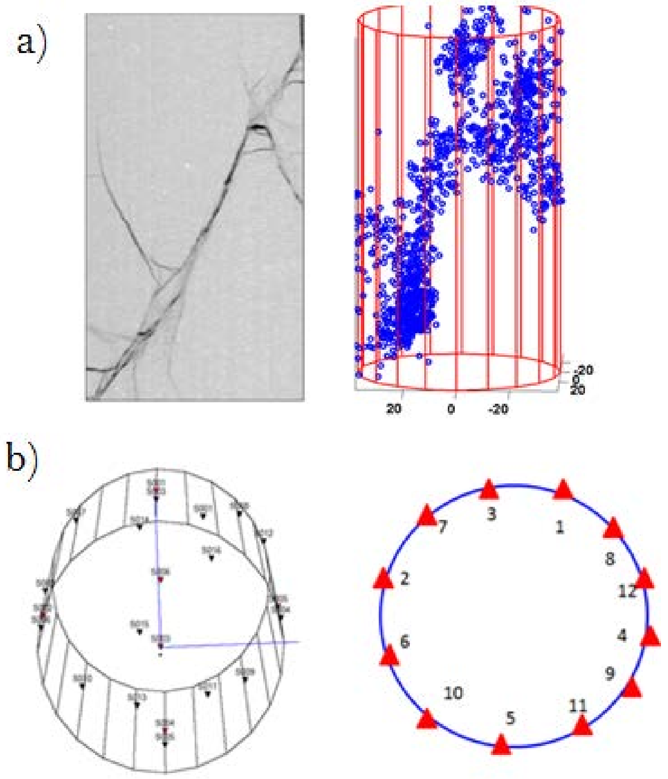

Figure S2|Studies on the Resistivity Function. (a) Final rough fault configuration (a slice of X-ray images) and the recorded events (blue dots) through one-loading cycle in **Lab.EQ2** [10]. b) Sensor -network mounted on the Basalt rock sample .The numbers indicates the sensors .

3. "Phase transition" in functional acoustic networks

Kibble–Zurek mechanism (KZM) - The *Kibble–Zurek mechanism (KZM)* describes the spontaneous formation of defects (symmetry breaking) in systems crossing a second-order (continuous) phase transition at a finite rate [14-15]. Experimental evidence of the KZM has been observed in superfluid ^{3}He [16], in superconducting films [17], in ion chains [18] and magnets or ferroelectrics with discrete microscopic symmetries [19]. Based on the KZM, in the vicinity of the critical point, the dynamics can be subdivided into the three main stages [14]: adiabatic, frozen, and again adiabatic (see Fig.S.3). Note that based on universality classes for second-order phase transitions, this mechanism provides a prescription for estimating a correlation length ξ and then, from this, the density of defects. For a second-order phase transition that evolves quasi-statically, ξ diverges at the critical point and no defects are formed [20].

The idea behind the KZM is to compare the relaxation time (or healing time of the system) with the timescale of change of the control parameter (i.e., ε). We assume a linear change of the control parameter in the vicinity of the critical point $\varepsilon(t) = t/\tau_s$, where τ_s is the ramp time. The relaxation or healing time $\tau(\varepsilon) = \frac{\tau_0}{|\varepsilon|^{vz}}$ determines the reaction time of the order (or state) parameter. Here, v and z are spatial and dynamical critical exponents, and τ_0 is a timescale set by microphysics. The system can adiabatically follow the change imposed by the local stress (or strain) ramp if relaxation time characterized by $\tau(\varepsilon)$ is outside interval $\hat{t} = (\tau_0 \tau_s^{zv})^{\frac{1}{1+vz}}$ around the transition point ($\hat{t}$ is freeze-out time where correlation length is effectively constant) [Fig.S.3a]. The system will cease to maintain with the imposed change at time $\hat{t}$ before reaching the critical point. The broken symmetry is chosen by fluctuations when their coherence length is given by [14-15]: $\hat{\xi} = \xi_0 (\tau_s / \tau_0)^{\frac{v}{1+vz}}$. According to the KZM, the choice of broken symmetry is random within fluctuating domains of this size. Topological defects (such as dislocations or vortices) are formed with the density of one defect fragment per domain. Here the idea is as follows: We probe the evolving ground state of the system in real time via acoustic (phononic) excitations, and then we access the strongly non-equilibrium dynamics directly, rather through its aftermaths.

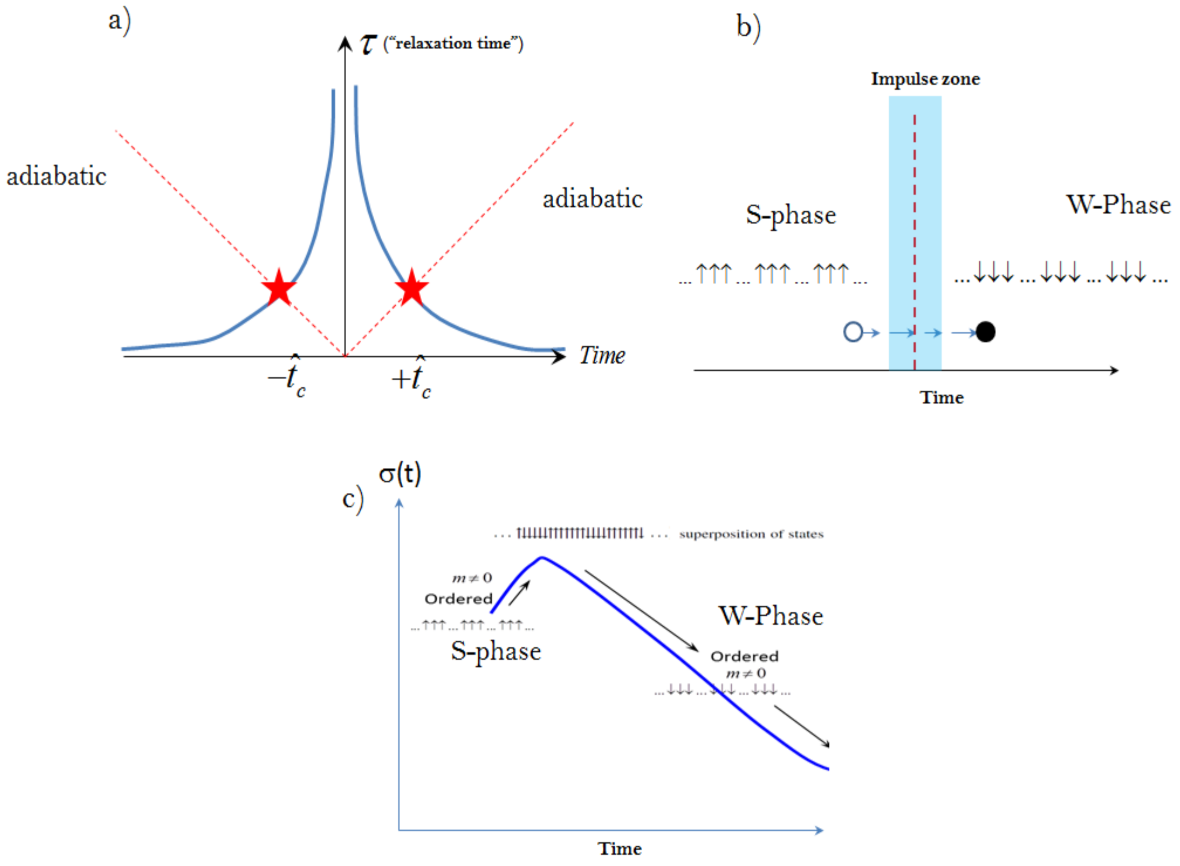

Figure S3. (a) For a continuous transition and around the transition point set at t=0, the relaxation time of the system diverges (adapted from [15]). (b,c) Transition from S-phase to fast Weakening regime (W-phase) is accompanied with nucleation of topological defects. Arrows indicate the direction of $s_i = sign(\frac{\partial k_i}{\partial t}); s_i = \{-1,1\}$ where we mapped acoustic emissions to K-strings and then the spin- system. c) Crossing critical point is interpreted to transition between two degenerate states. (Here we have half of the (a) where the system "pressure-quenched" from ordered phase to a disordered state).

To identify the signature of defects, we use the concept of undulating "K-strings". In Fig.S.4b-d, we show the evolution of K_{max}. The trends of evolution of K_{max} and R(*t*) are similar and include two main sub-stages separated by an inflection point: the first sub-stage is the accelerating portion and the second (nucleation) portion with negative concavity (Fig.S5-S.6).

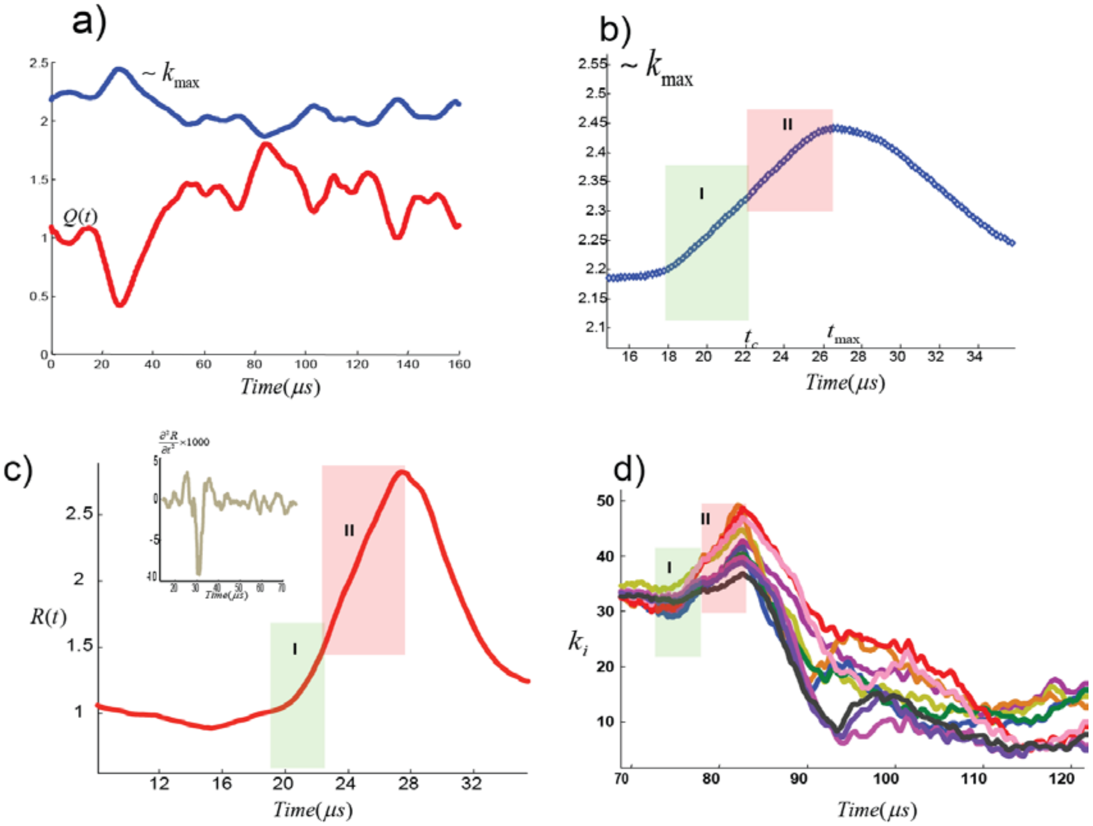

Figure S.4| a) Evolution of maximum degree of the constructed networks (K_{max} is the scaled value) and the modularity index (Q(t)) ;b) evolution of K_{max} around the impulse-zone. S-phase includes two sub-stages: accelerated/loading-zone (stage I) and nucleation zone (stage II). c) R-profile around the critical point. Inset shows the second derivative of R(t). (d) Evolution of node degree for an event from Lab.EQ2.

***K-strings (chains)*:** To visualize and study defects in the S-W transition, we use plot the node degree (k_i as the number of links attached to i th node) in a polar coordinate system $((r_i, \theta_i)_{i=1,...,Nodes}$ where $r_i = k_i$ and θ_i indicate the fixed position of each node around the cylindrical rock specimen. In other words, we map the cylindrical samples on a circle and evaluate the variation of $r = k$ on each position (node) while we consider the temporal evolution of each single event (Figs.S.7-8).

As shown in Figs S.7-8, the onset of a *"defect"* as the flipping of the curve normal does coincide with onset of divergence in the inverse of B.C. Also, this visualization confirms formation of kinks (more precisely kink-antikink pairs) in the course of the S-W transition which results in non-linear (i.e., plastic) behaviour. Since the trend of R(t) is similar to <K(t)> in terms of the temporal local maxima and minima and according to Fig.S.1. R(t) ~ $R(t) \sim \sigma(t)$, then as the result of empirical inference <K(t)> $\sim \sigma(t)$. Onset of non-linearity in <K(t)> , then, corresponds with beginning of non-linearity (plasticity) in $\sigma(t)$.

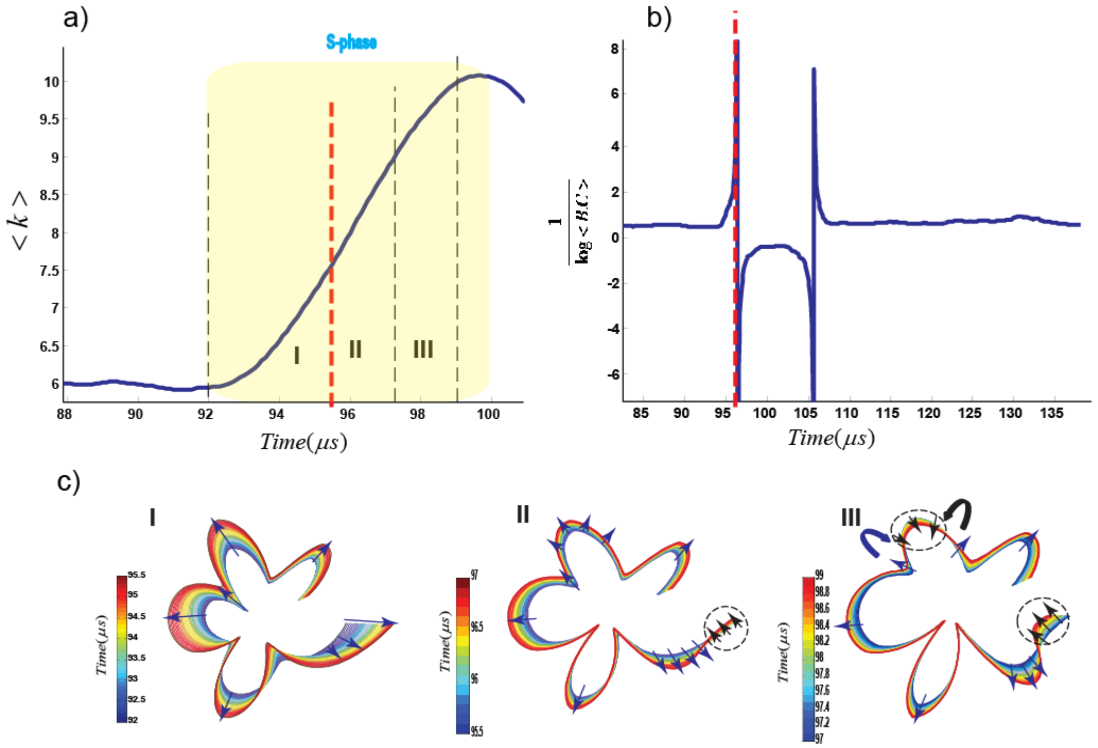

Figure S5. a) The mean number of links (<k>) versus time for event#24 from Lab.EQ4. b) Transition to "*precipitate –out* " or nucleation zone is imprinted in diverging the inverse of mean betweeness centrality (B.C). (c) We have shown accumulated 2D spatio-temporal patterns of nodes' degree in the polar system for each time interval as in panel (a). Transition from the linear stage (I) to the non-linear regime (II+III) is indicated by the onset of local defects (black arrows), inducing formation of kinks. The arrows are normal to strings and crumpled strings destroy long-range order in string normal. At the onset of the W-phase (~307μs) the dominant behavior of the system is governed by" defects".

The formation and merging of the kinks can be followed in Fig S.5. Interestingly, prior to the failure (defined as the point at which <k> reaches its maximum) and during nucleation of kinks, $A = \left(\max(\frac{\partial^2 k}{\partial x^2}) \right)_{t \in [-\tau_c, 0]}$ is nearly constant (Fig.S.6d). Then the maximum and minumum curvature of K-strings are nearly constant in $t \in [-\tau_c, 0]$ which again coincides with the left hand impulse in the inverse of B.C. (Fig.S.6b). This zone defines the nucleation time of defects and the nucleation rate can be estimated by considering the number of flipped nodes at the end of this precipitation-out zone (Kibble scenario: topological defects can form during ramp time). Please note that Zurek's *freeze-out* time occurs at the end of this phase (Fig.S14-S.15).

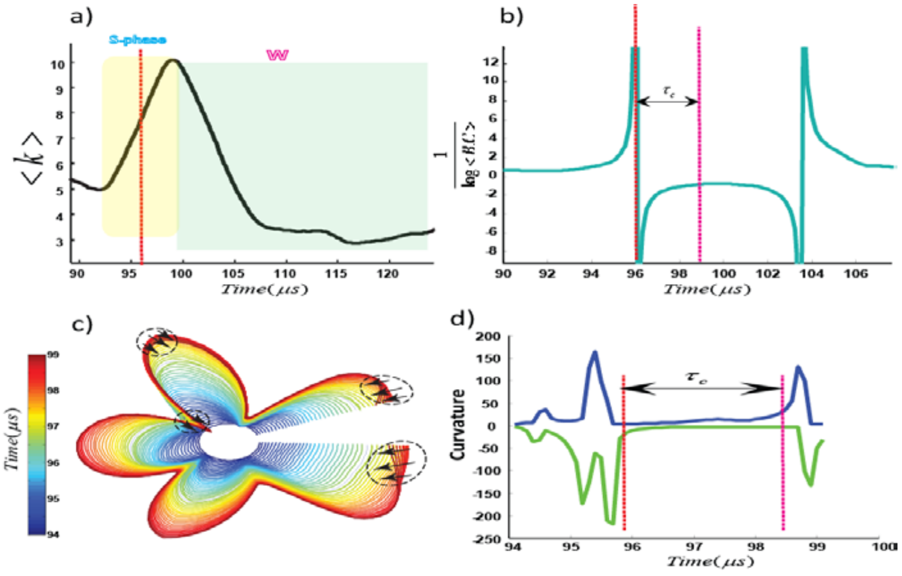

Figure S6. a) The mean number of links (<k>) versus time for event#101 from Lab.EQ2 with softening signature in S-phase. b) Transition to the nucleation zone is imprinted in the divergence of the inverse mean betweeness centrality (B.C). (c) Accumulated 2D spatio-temporal patterns of node degree in the polar system in impulse regime prior to W-stage. (d) Onset of impulse regime can be determined by the most stable evolutionary phase of diploes of critical curvatures of K-strings. The blue and green lines shows maximum positive and negative curvatures of K-strings ($\frac{\partial^2 k}{\partial x^2}$), respectively. The duration of the nearly stable phase is identical to the duration shown in panel (b),i.e., the nucleation time of defects.

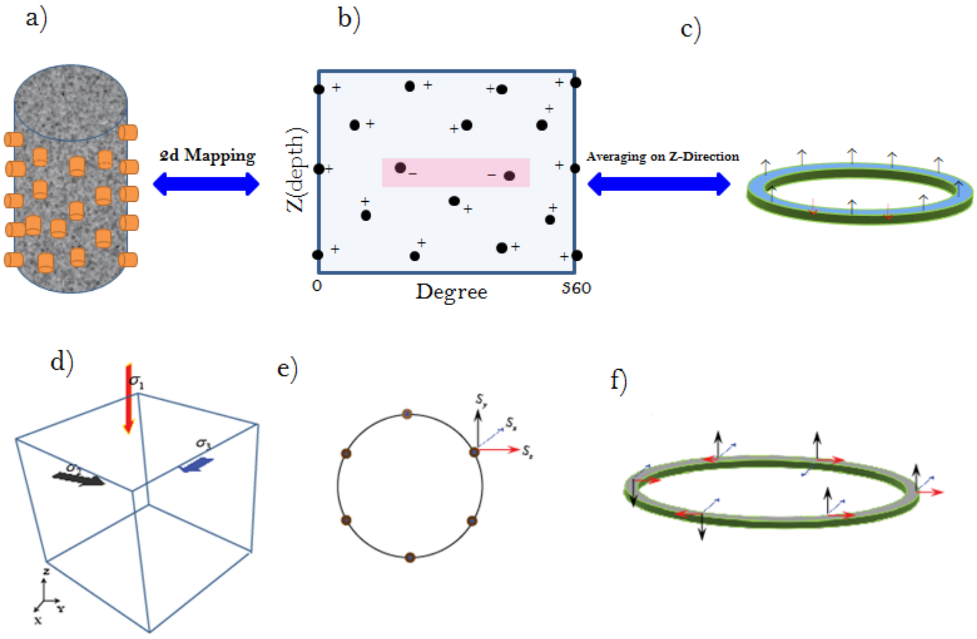

Figure S7. a-c) A 3d array of sensors on a surface of a cylindrical sample is mapped to a 2d configuration with assigning a direction (out or in) per each site . Furthermore, in a true 3D configuration (d-f) with using true triaxial tests [1] we can find out a 3D vector ‘components per each site.

According to KZM theory, the rate of transition to the critical regime where we observe the first defect (here the rate of “stress (pressure)-quench”) controls defect density. To measure the ramp rate, we carefully measure the slope of R(t). This will yield the arte of R(t) and from Fig.S1 we have $R(t) \propto \sigma(t)$ and then we obtain (local) stress rate. In this study, we assume a linear ramp.

Next, we measure the number of flipped nodes and then density of kinks for typical events (Fig.S.8-10). Interestingly, faster ramp (shorter ramp-time) shrinks the precipitate-out time (which is the time that takes to complete the phase transition) and increases the density of defects (Fig.S.9) which decreases $\hat{\xi}$ (coherence length)-also see Fig.S.11,S.14. We can fit an algebraic function like $\tau_c \propto (\frac{dR}{dt})_{t_c}^{-0.35}$ to nucleation times versus ramp-rate (Fig.S.9-10).

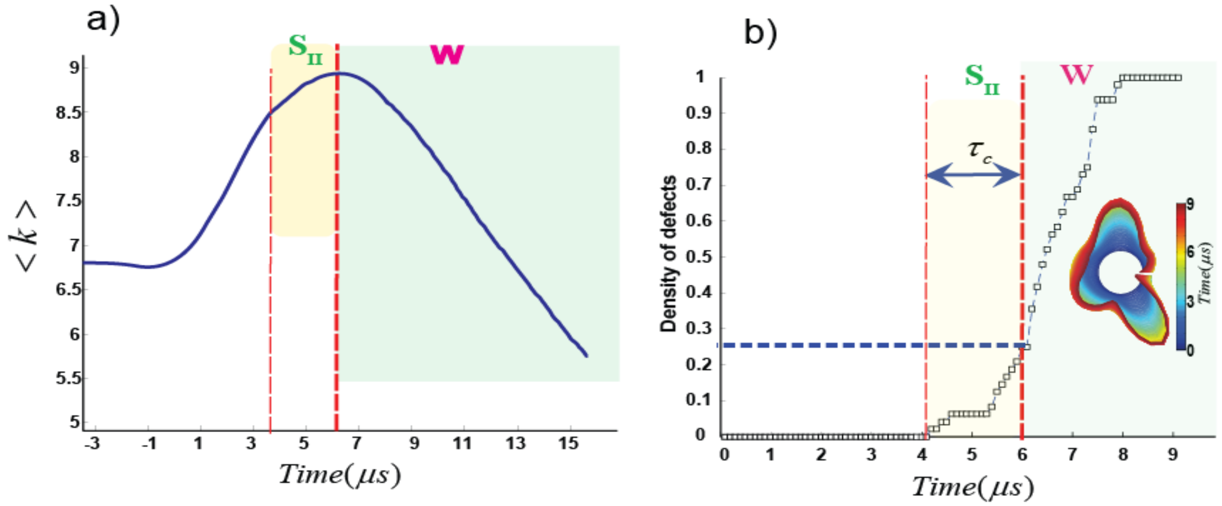

Figure S.8| Density of defects . Close to entering in to W-regime where <k> reaches to its maximum value, the fraction of flipped defects are calculated as defect density. The density of defects precisely are determined based on frozen-correlation length as we have shown in FigureS S.14-15. The time characteristic of nucleation of kinks is defined by *precipitate-out* time τ_c which is the time that takes to complete the phase transition.

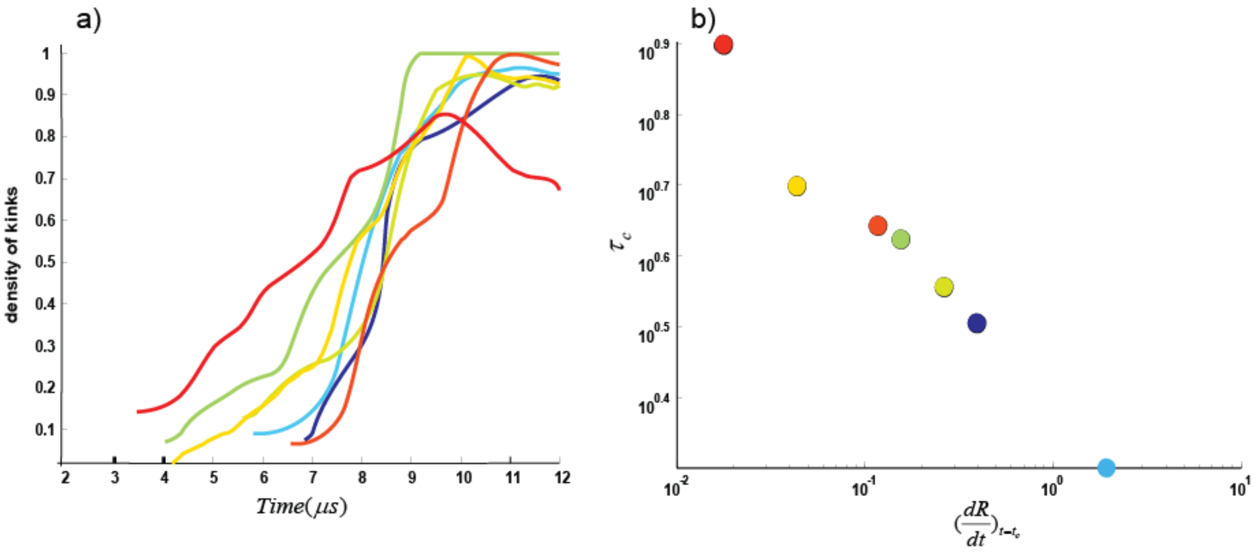

Figure S9. a) Density of defects versus time for 7 events from Lab.EQ 4. (b) Nucleation time versus linear ramp-rate for the same events.

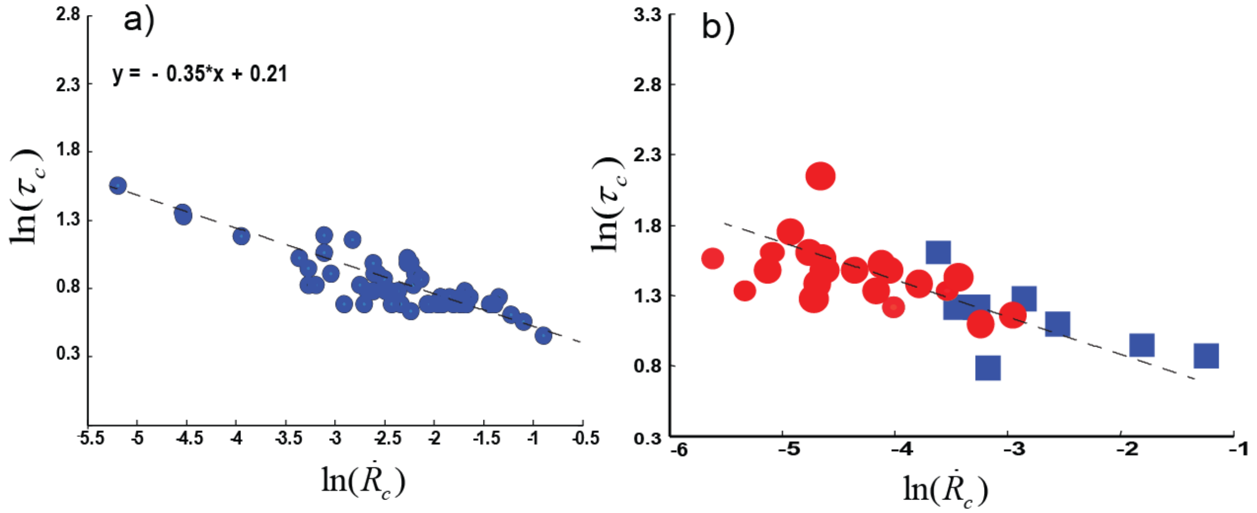

Figure S.10. Scaling relations for typical events from Lab.EQ (2 and 4); a) precipitate–out time versus ramp-rate : $\tau_c \propto (\frac{dR}{dt})_{t_c}^{-0.35}$. b) The faster ramp rate nucleation zone shrinks the nucleation time (τ_c in μs). The datasets are from Lab.EQ (1-red) and Lab.EQ(2-blue).

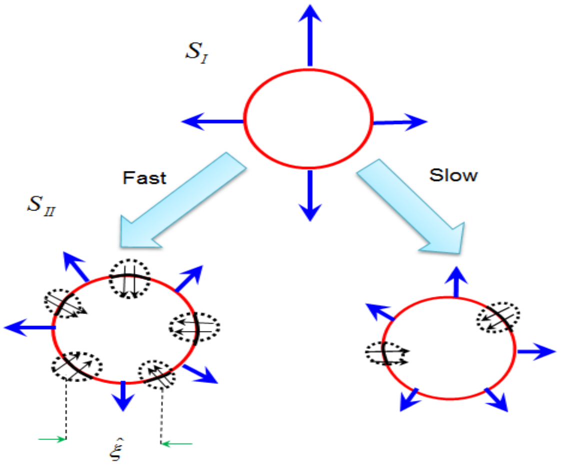

Figure S11. Faster loading rate increases the population of domains in K-strings. In this schematic figure, kinks are depicted by black segments and arrows. Slower transitions yields a smaller number of fragments.

Deformation of K-strings prior to the nucleation of kinks can be mapped on a spin-chain by considering $s_i = sign(\frac{\partial k_i}{\partial t}); s_i = \{-1,1\}$ [21]. With this mapping, defects are represented with negative S_i indicating flipped nodes (Fig.S.12), and *m* is the *order parameter* of the K-strings. According to KZM in the vicinity of the critical point the state of the system freezes ; m^2 and ξ are effectively fixed. The order parameter shows an impulsive trend and remains effectively frozen [27]. Furthermore, we have calculated a correlation function for the system including all nodes while it approaches the critical point (Fig.S13 a, b) . The correlation function has the form $G(x) \equiv (1-\frac{x}{L})\exp(-\frac{x}{\xi})$ where L is the total number of nodes, x is distance and ξ is the correlation length. Correlation length ξ is the cut-off length of the correlation function where for cases with shorter distance than the correlation length, a power law function can be fitted [20].

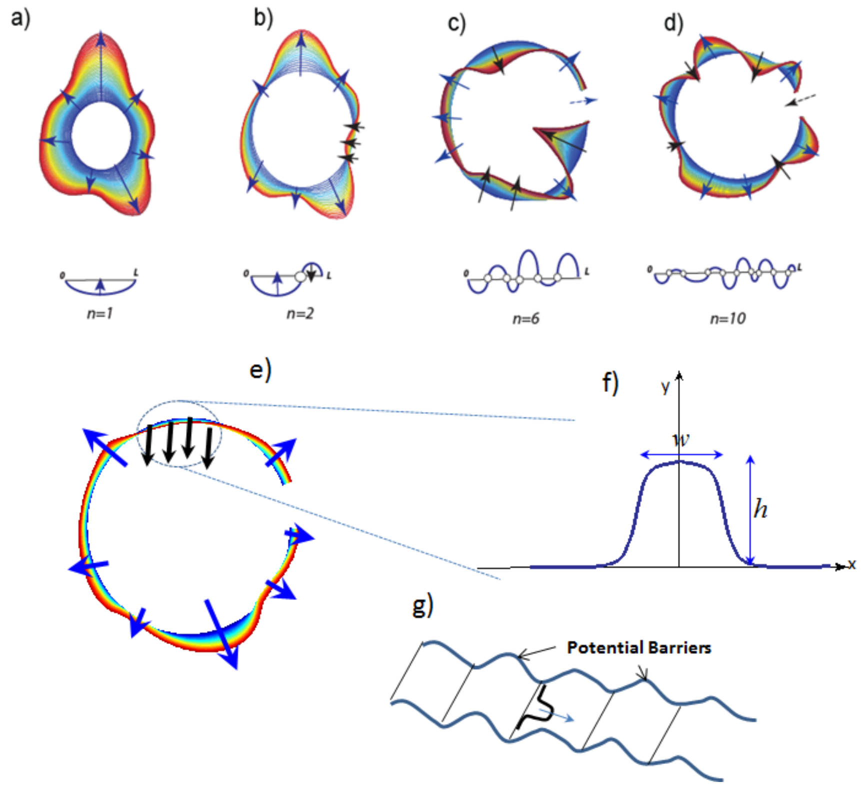

Figure S12. K-strings and normal modes. Patterns of a K-string can be mapped to a vibrating string with the length of L (i.e., number of nodes) with normal modes as the characteristic values (spectrum) of the K-string. Here, we show different time-snapshots of K-strings from our experiments. The panel (a) is a fully polarized state (fully ordered phase) with n=1 normal mode which, then, is the fundamental mode and panels (b-d) are strings with different defect zones representing "*overtone*" modes.(e-g) the nucleated defects can be considered as a kink-pair (with a simplified rectangular shape) which moves on a substrate potential in analogy with the Peierls potential [24-26].

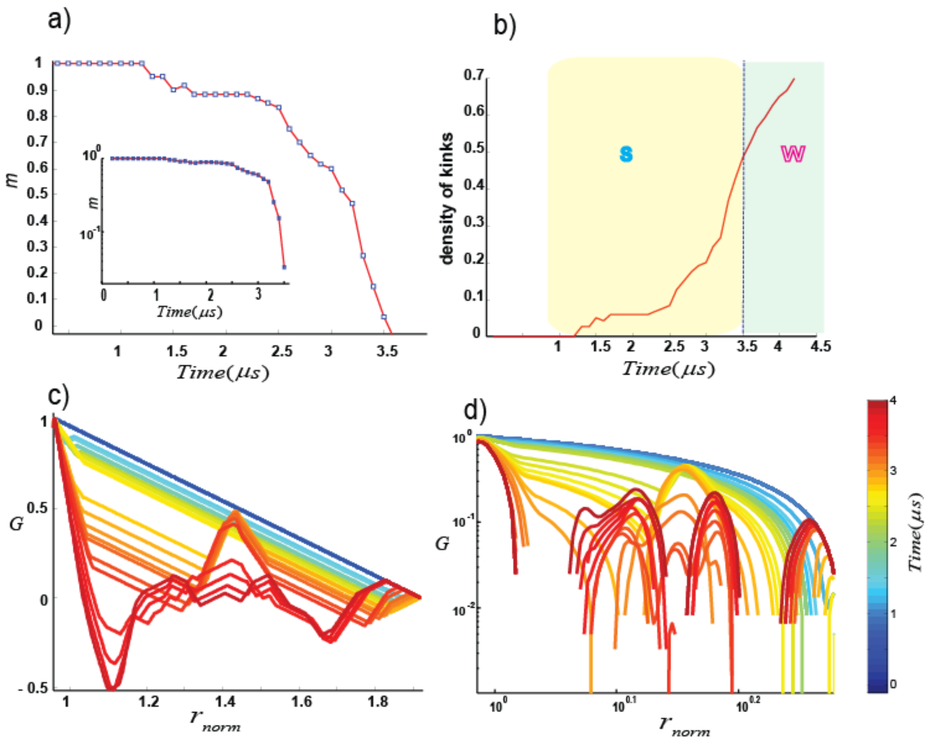

Figure S.13. Continuous transition from S to W Results from a cracking noise from Lab.EQ3 (a) order parameter as the average of $S_i = sign(\frac{\partial k_i}{\partial t})$ over nodes versus time when it approaches the critical point λ_c. (b) The corresponding density of kinks.(c,d) real-time correlation function .

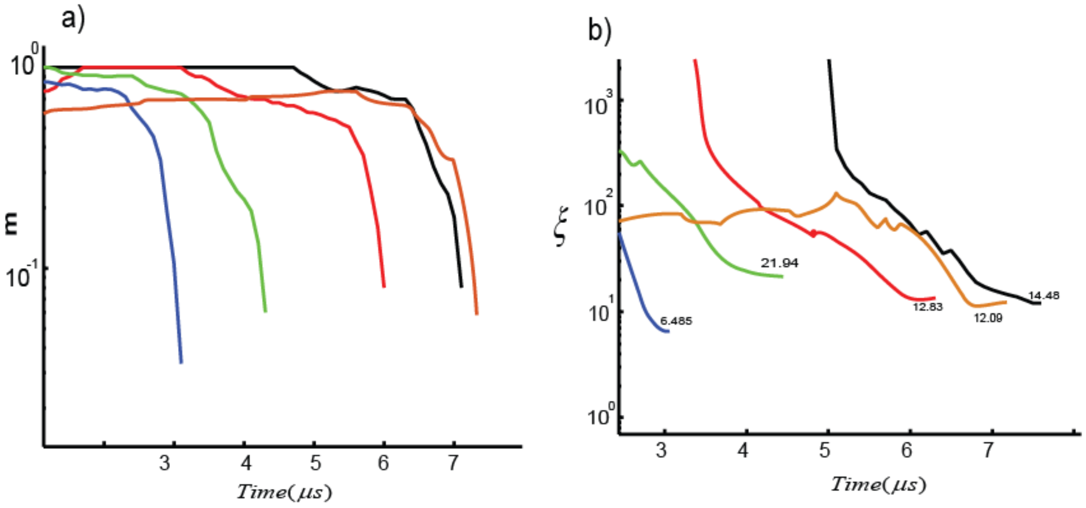

Figure S.14. Order parameter $m = < s >$ and correlation length ξ for five different events with different local ramp rate. The faster event (blue) imprints shorter "frozen" correlation length $\xi \approx$ 6.4 close to the transition point while the slower event (green) establish longer frozen correlation length $\xi \approx$ 21.9. The events are from Lab.EQ4. The size of the system is 300 nodes .

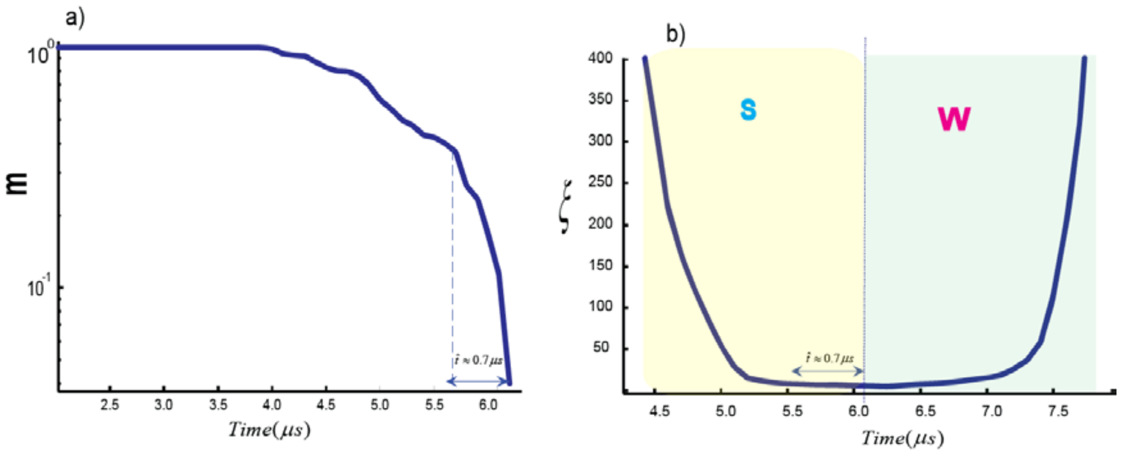

Figure S.15. Order parameter $m = < s >$ and correlation length ξ for event #102 from Lab.EQ2 with 300 nodes defining the network. Approaching the critical point, the correlation length is effectively frozen .The duration of this frozen zone is around ~ 0.7μs corresponding to KZ's freeze-out time.

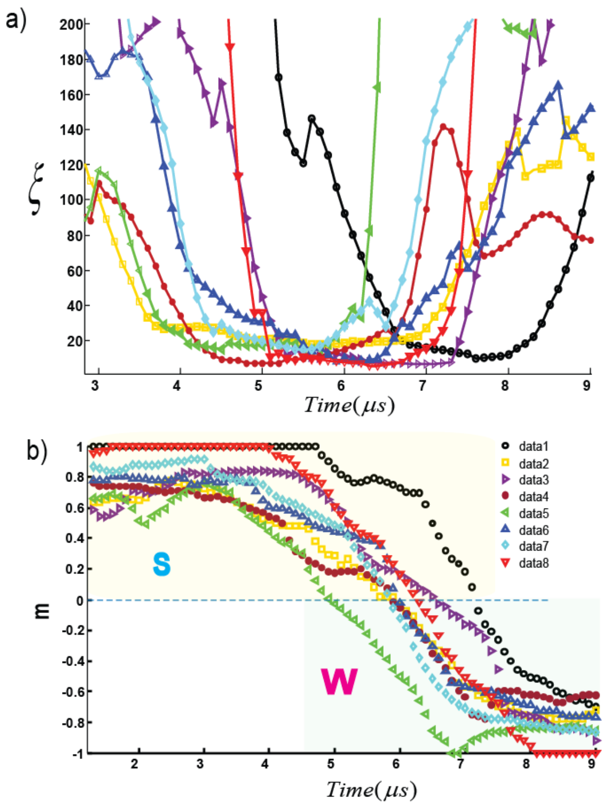

Figure S.16. Correlation length ξ and order parameter $m = < s >$ and for 8 events from Lab.EQ1 with 300 nodes defining the networks. Approaching the critical point (the S-W transition), the correlation length is effectively *frozen* (a) .

The evaluated events in our study do satisfy the condition of the second-order phase transition: under this class of phase transition, order parameter $m = < s >$ rises (or declines) from zero (or 1) continuously as shown in Fig.S.13-15 and Fig.S17.

Nevertheless, we could recognize some rare events that do not obey this classification (Fig.S.18): The order parameter jumps (drops) discontinuously to a non-zero value below R_{max} as the critical point. We will investigate the properties of this first-order crackling noises in future work.

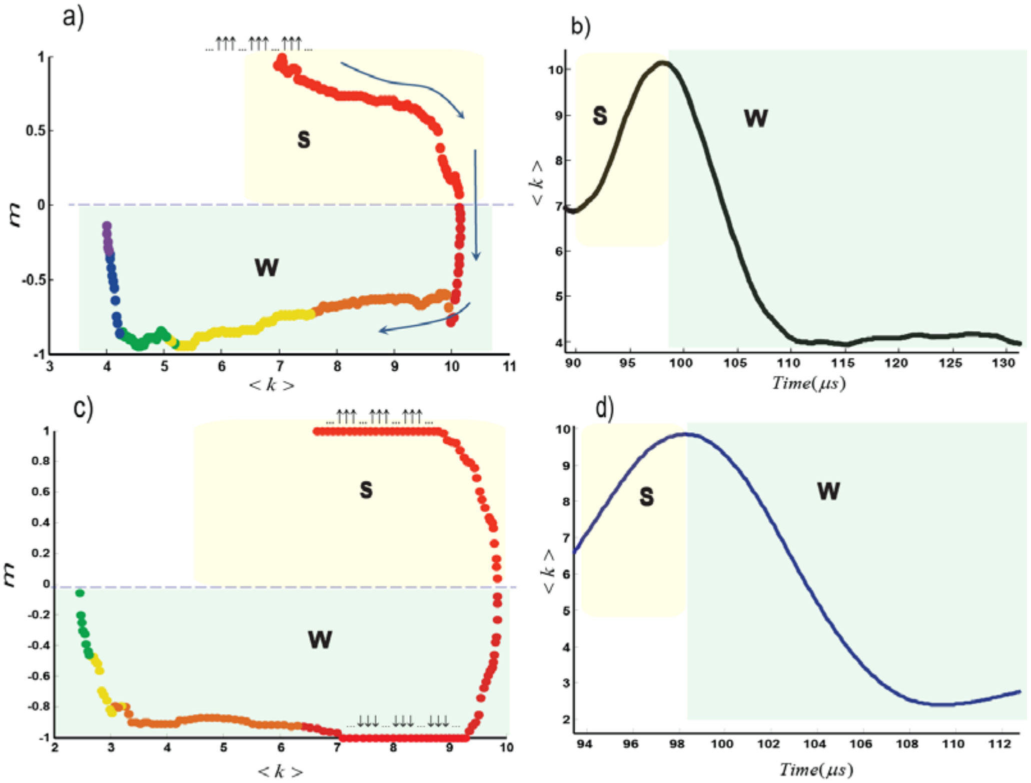

Figure S.17. a,b) Order parameter $m = < S >$ versus <k> for #42 from Lab.EQ3 and <k> versus time .(c,d) Event# 102 from Lab.EQ4

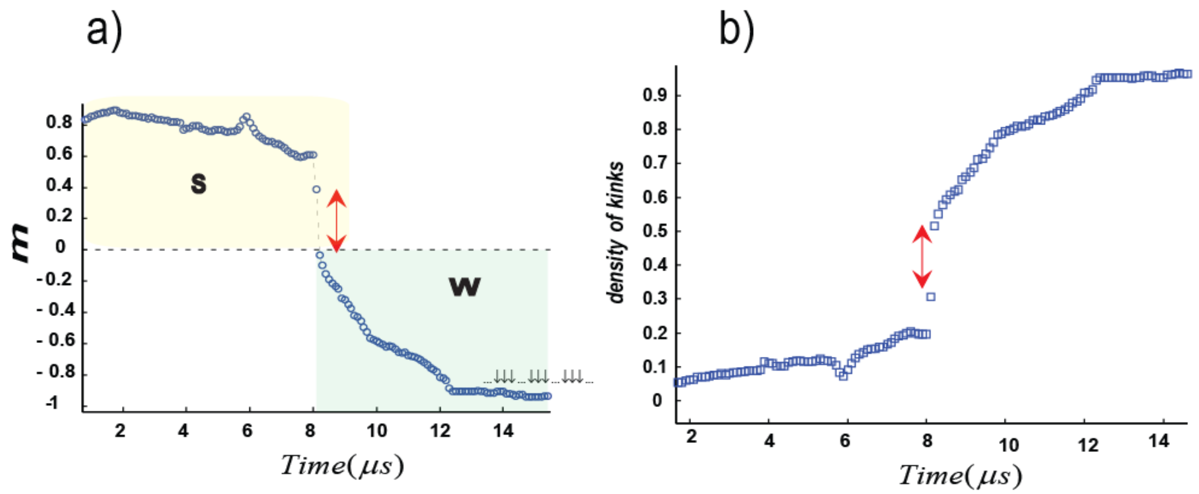

Figure S.18. Observation of the *first-order transition* in one of events(#402 in Dray-Basalt Experiment). In this rare event, the order parameter jumps discontinuously to a non-zero value below R_{max} as the critical point (failure).

Double KZM in R(t) : *Order-Disorder-Order* Transitions

Now, we can evaluate a time-reversal transition from the W to S phase while we approach the critical point from the right. Approaching the critical point λ_c from the left (from the S-phase to the W-phase – weakening scenario) or right (from the W-phase to the S-phase - a time reversal or healing scenario) results in slightly different characteristics of the frozen-zone. Note that all the above results were focused on the S-W transition. The rate of S-ramp (linear strengthening rate or local ramp rate) for most of the recorded events is higher than the rate of the W-ramp (i.e., linear weakening rate). As we showed, and according to KZM, slower ramp marks longer KZ's freeze-out time and then time-reversal approaching to the critical point λ_c yields longer freeze-out time : $\hat{t}_c^R \geq \hat{t}_c^L$.

Next we estimate the linear weakening rate. We note that the defect density from the S-phase is in balance with number of healed points when we are approaching from right to the critical phase. Using the KZM scaling law for defect density ρ, we have:

$$\rho_s \propto (\frac{\tau_s}{\tau_0})^{\frac{-2\nu}{1+\nu z}}$$

$$\rho_w \propto (\frac{\tau_w}{\tau_0})^{\frac{-2\nu}{1+\nu z}}$$

where τ_s , τ_w are the ramp-times for S and W phases, respectively. At $\varepsilon = 0$ (failure point), we approximate : $\rho_w \cong 1-\rho_s$ and then we get: $\tau_w \propto \tau_0[1-(\frac{\tau_s}{\tau_0})^{\frac{-2\nu}{1+\nu z}}]^{\frac{1+\nu z}{-2\nu}}$, in which τ_0 is determined by the microscopic details of the system [14-15]. Therefore, the linear weakening rate is given by: $(\frac{dR}{dt})_w \propto \tau_0[1-(\frac{\tau_s}{\tau_0})^{\frac{-2\nu}{1+\nu z}}]^{\frac{1+\nu z}{2\nu}}$: A faster local S-ramp inversely scales with *weakening rate* . This is an important result since it has been shown that weakening rate is correlated with the global *rupture velocity* of cracks [1, 22-23].

References

[1] Ghaffari, H. O., Nasseri, M. H. B., & Young, R. P. (2014). Faulting of Rocks in a Three-Dimensional Stress Field by Micro-Anticracks. *Scientific Reports*, 4.

[2] Ghaffari, H. O., Thompson, B. D., & Young, R. P. (2014). Complex networks and waveforms from acoustic emissions in laboratory earthquakes. *Nonlinear Processes in Geophysics*, 21(4), 763-775.

[3] Iwayama, K. et al. Characterizing global evolutions of complex systems via intermediate network representations. *Scientific Reports* 2, 423 (2012).

[4] Ghaffari, H. O. & Young, R. P. Topological complexity of frictional interfaces: friction networks. *Nonlinear Processes Geophys*. 19, 215 (2012).

[5] Newman, M. E. J. & Girvan, M. Finding and evaluating community structure in networks. *Phys. Rev. E* 69, no. 026113 (2004).

[6] Newman, M. E. J. *Networks: An Introduction* (Oxford University Press, 2010).

[7] Blondel, V. D., Guillaume, J. L., Lambiotte, R., & Lefebvre, E. (2008). Fast unfolding of communities in large networks. *Journal of Statistical Mechanics: Theory and Experiment*, 2008(10), P10008.

[8] Ben-David, O., Rubinstein, S. & Fineberg, J. Slip-Stick: The evolution of frictional strength. *Nature*. 463, 76 (2010).

[9] Ghaffari, H. O., & Young, R. P. Acoustic-friction networks and the evolution of precursor rupture fronts in laboratory earthquakes. *Scientific Reports*, 3. (2013).

[10] Thompson, B. D., Young, R. P. & Lockner, D. A. Premonitory acoustic emissions and stick-slip in natural and smooth-faulted Westerly granite. *J Geophys Res*. 114, B02205J (2009).

[11] Thompson, B.D,R.P. Young, D.A. Lockner, Fracture in Westerly Granite under AE Feedback and Constant Strain Rate Loading: Nucleation, Quasi-static Propagation, and the Transition to Unstable Fracture Propagation. *Pure and Applied Geophysics*, 163 (5-6): 995-1019 2006.

[12] Benson, P. M., Vinciguerra, S., Meredith, P. G., & Young, R. P. Spatio-temporal evolution of volcano seismicity: A laboratory study. *Earth and Planetary Science Letters*, 297(1), 315-323 (2010)..

[13] Benson, P. M., Vinciguerra, S., Meredith, P. G., & Young, R. P. Laboratory simulation of volcano seismicity. *Science*, 322(5899), 249-252. (2008).

[14] Zurek, W. H. Cosmological experiments in superfluid helium? *Nature* 317, 505–508 (1985).

[15] del Campo, A., & Zurek, W. H. (2014). Universality of phase transition dynamics: Topological defects from symmetry breaking. *International Journal of Modern Physics* A, 29(08).

[16] Bäuerle, C., Bunkov, Y. M., Fisher, S. N., Godfrin, H. & Pickett, G. R. Laboratory simulation of cosmic string formation in the early Universe using superfluid 3He. *Nature* 382, 332–334 (1996).

[17] Carmi, R. & Polturak, E. Search for spontaneous nucleation of magnetic flux during rapid cooling of YBa2Cu3O7−δ films through Tc. *Phys. Rev. B* 60, 7595–7600 (1999).

[18] Pyka, K., et.al Topological defect formation and spontaneous symmetry breaking in ion Coulomb crystals. *Nature communication*s, 4(2013).

[19] Chae, S. C. et al. Direct observation of the proliferation of ferroelectric loop domains and vortex-antivortex pairs. *Phys. Rev. Lett*. 108, 167603 (2012).

[20] Navon, N., Gaunt, A. L., Smith, R. P., & Hadzibabic, Z. Critical dynamics of spontaneous symmetry breaking in a homogeneous Bose gas. *Science*, 347(6218), 167-170(2015).

[21] Chaikin, P. M. & Lubensky, T. C. Principles of Condensed Matter Physics (Cambridge Univ. Press, 2000).

[22] Latour, S., Schubnel, A., Nielsen, S., Madariaga, R., & Vinciguerra, S Characterization of nucleation during laboratory earthquakes. Geophys. Res. Lett. 40(19), 5064-5069. (2013).

[23] Buehler, M. J., & Gao, H. (2006). Dynamical fracture instabilities due to local hyperelasticity at crack tips. *Nature*, 439(7074), 307-310.

[24] Braun, Oleg M., and Yuri S. Kivshar. *The Frenkel-Kontorova Model: Concepts, Methods, and Applications* (Springer Science & Business Media, 2004).

[25] Bohlein, Thomas, Jules Mikhael, and Clemens Bechinger. "Observation of kinks and antikinks in colloidal monolayers driven across ordered surfaces." *Nature materials* 11, no. 2 (2012): 126-130.

[26] Eshelby, J. D. "The interaction of kinks and elastic waves." In Proceedings of the Royal Society of London A: Mathematical, Physical and Engineering Sciences, vol. 266, no. 1325, pp. 222-246. The Royal Society, 1962.

[27] Liu, Cheng-Wei, Anatoli Polkovnikov, and Anders W. Sandvik. Dynamic scaling at classical phase transitions approached through nonequilibrium quenching. *Phys. Rev. B* **89**, 054307 (2014).
